# A Rare Case of Giant Cell Tumour of Tendon Sheath and Its Arthroscopic In Toto Excision

**DOI:** 10.7759/cureus.50365

**Published:** 2023-12-11

**Authors:** Ashwin Deshmukh, Rachit Mitra, Ishan Shevate, Rahul Salunkhe

**Affiliations:** 1 Orthopaedics and Trauma, Dr. D. Y. Patil Medical College, Hospital and Research Centre, Pune, IND

**Keywords:** arthroscopic resection, arthroscopic surgery, minimal invasive approach, gct, giant cell tumors, tenosynovial giant cell tumor, tendon sheath, anterior cruciate ligament (acl), acl

## Abstract

The aim of this study is to bring attention to a unique occurrence in an uncommon location and to describe our approach to treatment in this context. We describe a case of a 36-year-old male who presented with complaints of pain in his left knee for three months, with a restricted range of motion, without a prior history of trauma. A thorough knee examination was performed, which was unremarkable except for a restricted range of motion and tenderness along the medial joint line. A plain radiograph of the knee revealed no bony injury. MRI was done to assess the extension and it confirmed a soft tissue mass beneath the patella.

The patient was taken up for surgery after a pre-anesthetic checkup and the mass was removed arthroscopically in toto using a higher accessory antero-medial portal. The mass was removed with the help of a spatula without damaging it and sent for histopathological analysis. Histopathology confirmed that it was a giant cell tumour of the tendon sheath. The procedure was uneventful, and the patient achieved a full range of motion post-operatively.

## Introduction

The giant cell tumour of the tendon sheath (GCTTS) originates via the synovial lining of a joint, bursa, or the sheath adjacent to a tendon, and it is a non-cancerous growth [1-3]. The tendon sheath, bursa, and synovium are anatomical structures susceptible to developing neoplastic changes. These transformations are typically non-cancerous proliferation, with metastatic occurrences being rare [4-6]. While GCTTS is frequently observed in the fingers, its occurrence in the knee is exceptionally uncommon [5]. The uncertain origins of GCTTS encompass factors such as inflammation, clonal chromosomal abnormalities, and aneuploidy. The characteristic of this tumour entails the proliferation of synovial-like cells and deposits of hemosiderin [2,4].

## Case presentation

This is a case of a 36-year-old man complaining of pain in the left knee joint for three months, which was insidious in onset, mild in intensity, dull aching in character, continuous in nature, initiated at flexion, and aggravated in terminal flexion, range of movement (ROM) is 5-100 degrees of the knee. ROM with 5 degrees of extension lag and active flexion was restricted at 100 degrees and passive up to 140 degrees (painful) and was associated with mild knee swelling. A thorough clinical examination of the knee did not reveal any signs of instability or any meniscal injury.

**Figure 1:**
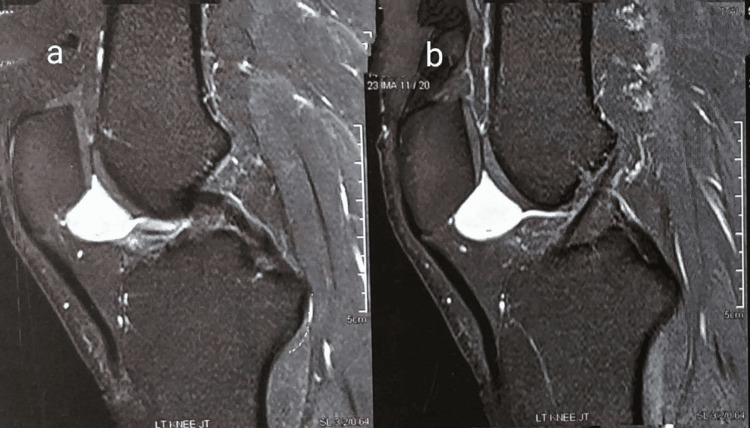
MRI images of the left knee joint A well-defined globular lesion appearing intermediate to hypointense on T1, showing avid enchantment, was noted in the infra-patellar recess within the Hofa's fat pad and abutting the inferior-posterior (non-articular) surface of the patella. It shows a thin linear extension arising from the anterior surface of the Anterior Cruciate ligament - suggestive of neoplastic etiology.

**Figure 2:**
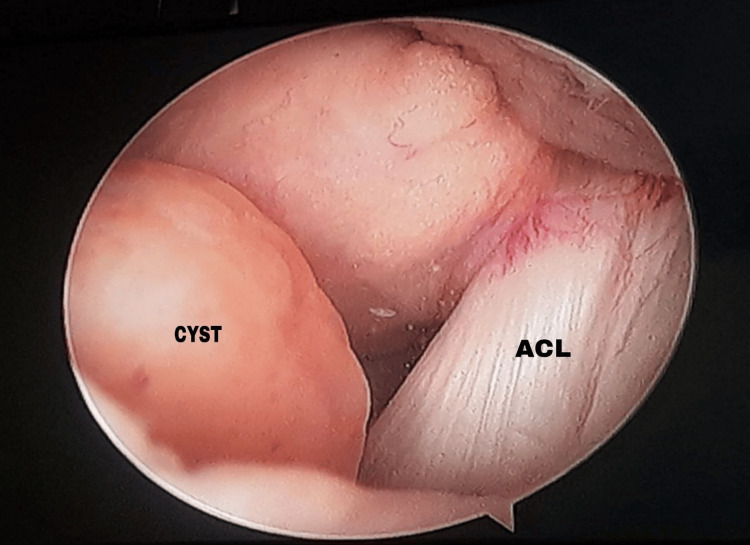
Diagnostic arthroscopy image The image shows an arthroscopic picture of the cyst at the superior surface of the medial meniscus.

An MRI revealed an intra-articular soft tissue cyst in the retro-patellar region (Figures 1a, 1b), with the differential diagnosis being a ganglion or a cystic lesion, while diagnostic arthroscopy demonstrated a mass located at the menisco-capsular junction of the medial meniscus (Figure 2).

**Figure 3:**
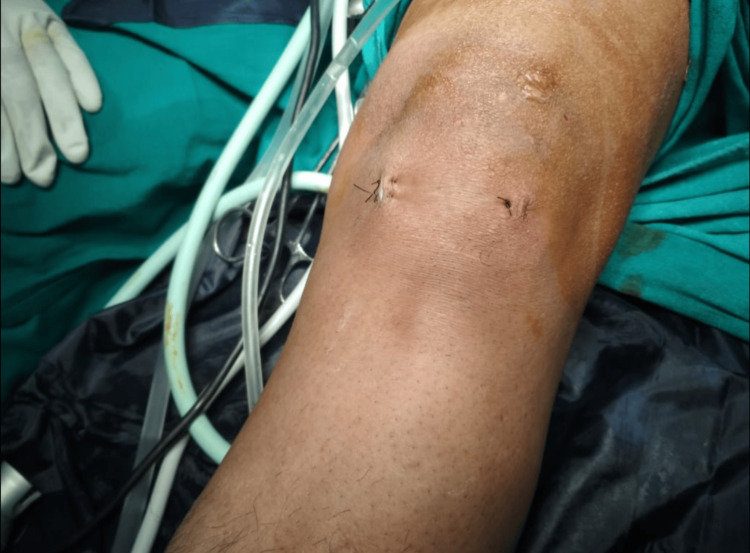
Accessory antero-medial incision, 10-15 mm far from the standard antero-medial incision

**Figure 4:**
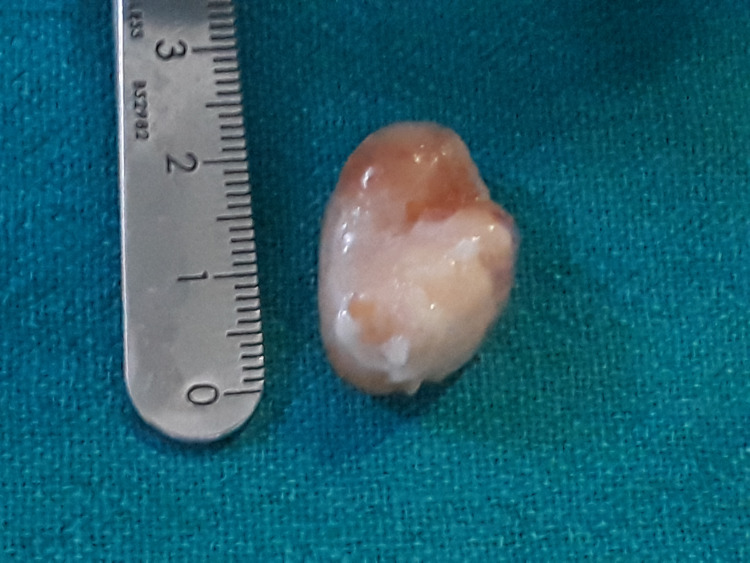
Excision of mass in toto

The mass was surgically removed through an all-arthroscopic, standard two-portal technique done using an accessory antero-medial incision and spatulas (used traditionally for introducing all inside implants of the meniscal repairs) to avoid rupturing the mass, 2*2 cm in size, and in-toto removal (Figures 3, 4). A standard antero-medial incision was avoided as the mass was seen too close to the insertion of the anterior horn of the medial meniscus; thus, standard antero-medial portal placement would have ruptured the cyst. The cyst was dissected carefully using an arthroscopic probe and shaver and avoiding any sharp instruments so as to avoid rupturing the cyst. Once the cyst was dissected, we introduced arthroscopic spatulas, these spatulas are used to introduce all-inside meniscal repair devices, which avoided any cartilage scuffing or damage and also acted as a guide for the instruments. Using two spatulas the cyst was delineated and separated from its attachments, delivered out from the accessory antero-medial portal, and sent for histopathological analysis.

**Figure 5:**
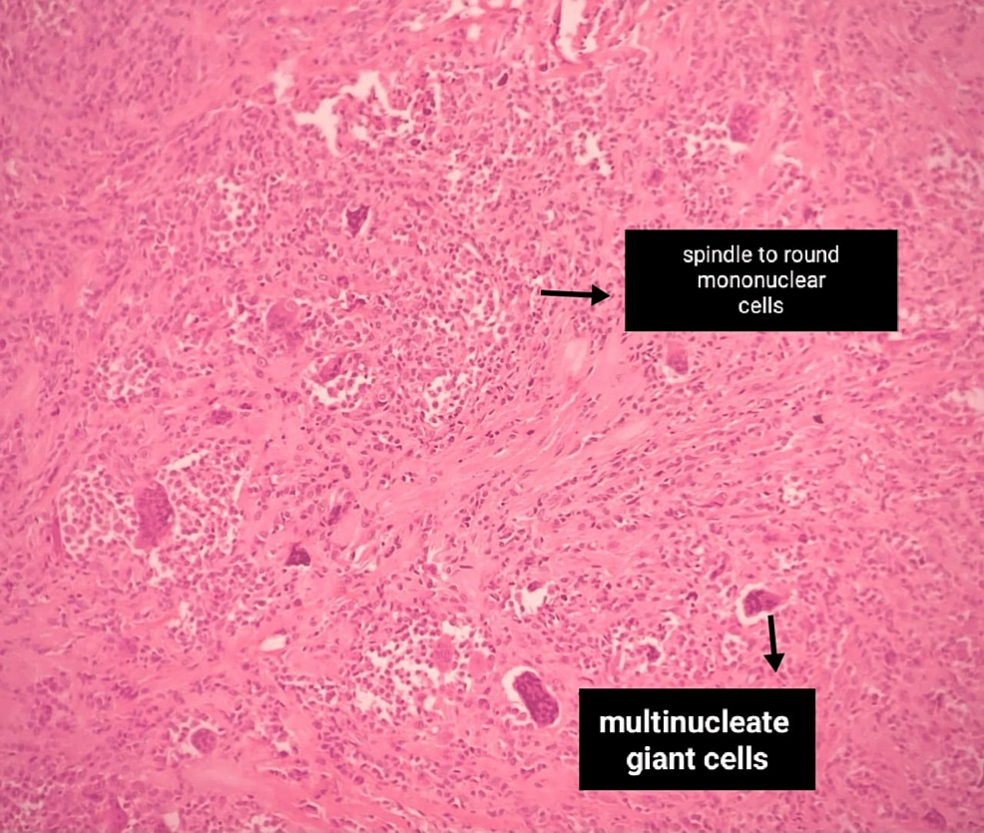
Histopathology image Histopathology slide of the mass excised shows spindle to round mononuclear cells and multinucleate giant cells.

According to the histopathological analysis, the micro section showed a well-circumscribed, lobulated, partly encapsulated mass composed of mononuclear cells interspersed with scattered, varying-size multinucleate giant cells (Figure 5). The tumour was seen at the peripheral resection margin and confirmed to be GCTTS (giant cell tumour of the tendon sheath).

## Discussion

GCTTS is a non-cancerous tumor originating in the synovial tissue of joints, tendons, or bursa. Tendon sheath lesions can exhibit intra or extra-articular growth, being either localized or diffuse. In the majority of instances, it appears as a localized variant of the tenosynovial giant cell tumor, primarily found in the fingers [2-4]. Typically, the diagnosis is established following an investigation prompted by suspicion [7,8]. Among the infrequent occurrences of extra joint involvement, the knee joint is the most commonly affected [3-5]. While GCTTS can develop at any age, it's most often observed between 30 and 50 years, with a higher occurrence among females in a 2:1 ratio [2,3]. This tumor was previously identified as extra-articular pigmentary villo-nodular synovitis (PVNS) due to its resemblance in radiological characteristics [6]. Typically, the common presentation involves gradual knee swelling, often accompanied by minimal to mild pain, and there is rarely a history of trauma, which closely resembles our clinical case [3,5]. MRI serves as the gold standard for diagnosing masses located in or around the knee joint [4,5]. The tumor typically exhibits reduced signal intensity on both T1 and T2 imaging [9]. The preferred treatment method is local excision performed through arthroscopy or arthrotomy [1,3,5].

In our case, a 36-year-old male presented with a history of pain and restricted range of motion of the left knee joint for three months with no significant history of trauma or injury. Clinically there was no muscle wasting. There was pain on complete extension and tenderness over the anteromedial and medial joint line with a negative Mcmurray's test and no signs or tests positive for antero-posterior or medio-lateral instability. On the basis of clinical examination, the patient was diagnosed with a meniscal pathology. In X-ray, there was no bony injury or no reduction in joint space. The patient was subjected to an MRI of the involved knee, which showed a single, well-lobulated hypointense cystic swelling in the inferior aspect of the patella, posterior to the patellar tendon. The MRI report suggested the swelling to be a ganglion.

The patient was taken up for diagnostic arthroscopy and excision of the ganglion. In cases with such presentation and swelling, care is to be taken while placing the standard arthroscopic portals. The standard antero-lateral portal is the primary viewing portal which is taken blindly following palpation of a parapatellar soft spot. After securing the antero-lateral portal, a needle is passed through the palpable medial parapatellar soft spot, and its intra-articular entry through the camera is visualized for the placement of the antero-medial portal. In our case, after introducing the scope from the antero-lateral, the swelling was noted very close to the medial parapatellar region at the surface of the medial meniscus anterior horn. Placing the needle for the antero-medial portal was crucial to make sure not to invade or pierce the swelling, following which, in case of a ganglion, the cystic fluid may get leaked in the joint, decompressing the swelling and making it difficult to demarcate the margins of the cyst which may lead to incomplete excision of the swelling.

Considering the location of the swelling, we introduced the needle from the far accessory antero-medial (AAM) portal, which is located 10-15 mm far from the standard antero-medial portal. After taking the skin incision, we introduced the probe for a round of diagnostic arthroscopy and probed the swelling from its margins. After probing, the 3.5 mm shaver was introduced and careful dissection of the swelling was performed. After dissection, an arthroscopic spatula was introduced from the AAM portal (after widening the portal). A large-sized artery forceps was introduced over the spatula, and the swelling was secured and held with the help of the forceps and carefully excised over the spatula. The arthroscopic spatula is an instrument that is used for the insertion and placement of all arthroscopic meniscal repair devices in cases of meniscal repairs. The 'U' shaped wide design of the spatula makes the passage of the arthroscopic instruments smooth, making sure to avoid any soft tissue engagement. The swelling was excised completely, maintaining its morphology, and the specimen was sent for histopathological examination (HPE).

The HPE described the swelling as a well-circumscribed, lobulated, partially encapsulated mass composed of mononuclear cells. Mononuclear giant cells have round to oval to spindled nuclei, with nuclear grooves, granular chromatin, and a small nucleus. Mitosis were present (10/10 HPF). Multinucleate giant cells exhibited 3-35 nuclei. There were varying amounts of collagenous stroma with hyalinization. Macrophages were present and metaplasia was not seen. A tumour was seen at the peripheral resection margin. Nuclear pleomorphism or abnormal mitosis was not seen. Focal ischemic necrosis was seen. The final impression of the HPE was suggestive of a giant cell tumor. Post-operatively the patient was followed for any residual or similar complaints to watch for relapse, which was uneventful at one-year follow-up.

## Conclusions

GCTTS are benign growths that can exhibit aggressive behaviour due to their infiltration of vital structures, making complete removal challenging. Knee MRI is a diagnostic tool, and curative treatment involves full excision, with rare instances of recurrence. Diagnosis is based on clinical and radiological criteria, and arthroscopy is the chosen treatment option post-diagnosis, with the accessory antero-medial (AAM) portal being used to avoid rupturing the cyst which may usually be fluid-filled (ganglion) or any hard swelling. Skillful use of arthroscopic instruments can facilitate complete in toto excision of the swelling, which can avoid the use of large incisions or open arthrotomy, which helps in reducing the length of the stay in the hospital, early mobilization, and moreover, minimizing the chances of infection rate as compared to cases of open surgeries or arthrotomy.
